# Supplementary material for: The Role of Immunohistochemistry as a Surrogate Marker in Molecular Subtyping and Classification of Bladder Cancer
Source: Diagnostics (Basel). 2024 Nov 8;14(22):2501. doi: 10.3390/diagnostics14222501 (PMC11592502; doi:10.3390/diagnostics14222501)
Supplement: Supplementary file 1 [file diagnostics-14-02501-s001.zip › diagnostics-3281320-supplementary.pdf]

Supplementary Table S1. Findings from main studies with immunohistochemistry as a surrogate marker

Main studies	NMIBC/MIBC	Number of patients	Molecular Subtypes Analysed	IHC markers	Main Findings	Conclusion
Terlevic R et al. (2023) [49]	MIBC	92	LumP, Luminal Unstable, Ba/sq	GATA3, KRT5/6, p16	Luminal and basal subtypes correlated with OS	Simplified molecular subtyping is feasible and may influence treatment selection. The simplified IHC panel is a promising tool for molecular subtyping of MIBC.
Goutas D et al. (2023) [54]	MIBC	77	Luminal , Basal	GATA3, KRT5/6, PDL1	Luminal (KRT5/6 -, GATA3+), Basal (KRT5/6+, GATA3 -). Strong association of PD-L1 expression with basal subtype	The basic IHC panel (KRT5/6, GATA3) is effective for molecular subtyping and identifying likely responders to PD-L1 therapy. Basal subtype patients may respond better to anti-PD-L1 therapy, suggesting potential stratification for immunotherapy.
Sanguedolce F et al. (2023) [55]	MIBC	92	Basal, Luminal, DP, DN	GATA3, KRT5/6, KRT20	Significant association between muscularis propia invasion patterns and molecular subtypes. No consistent differences in survival between subtypes.	he use of proper IHC markers is a cost-effective method for routine molecular subtyping of MIBC, with potential implications for treatment strategies.
Hesswani C et al. (2023) [48]	MIBC	93	Basal, GU, URO, Unclassified	GATA3, KRT5/6, p16	Subtyping feasible in most patients treated with radiotherapy; no significant association between subtypes and survival outcomes.	IHC-based molecular subtyping is feasible for MIBC in patients treated with radiotherapy, but it does not predict oncological outcomes; further prospective studies are needed.
Helal DS et al. (2023) [72]	MIBC	60	Luminal, basal, p53WT	GATA3, KRT5/6, p53, HER2, EGFR	Luminal subtype had the best OS and DFS; Basal subtype showed better NAC response; p53-WT was chemoresistant	Molecular subtyping using IHC is effective in predicting prognosis and treatment response. HER2 and EGFR are potential therapeutic targets.
Höglund M et al. (2023) [47]	NMBC/MIBC	455	UroA, UroB, UroC, GU, Ba/Sq, Sc/NE	UroA: KRT5, CDH3, EGFR<br>UroB: KRT5, CDH3, EGFR, GATA3, PPARG<br>UroC: KRT5 (with thinner and often interrupted layers)<br>GU: Negative for KRT5, CDH3; Positive for ERBB2, high CCNB1 expression<br>Ba/Sq: KRT5, KRT14, CDH3, EGFR<br>Sc/NE: ENO2, CHGA, SYP, negative for PPARG, GATA3, KRT20	The study emphasises the importance of aligning gene expression profiles with IHC-based cancer cell phenotypes. It identified that different cancer cell subtypes can exhibit similar gene expression profiles, and that gene expression clustering can be influenced by non-cancerous cell infiltration and proliferation, leading to divergence and convergence phenomena	The LundTax classification system effectively harmonises gene expression profiling with cancer cell phenotype classification obtained through IHC analysis, allowing for a more accurate and stage-independent classification of urothelial carcinomas. This system is applicable to both NMIB, and MIBC cancers, providing a solid foundation for future clinical applications.
Ying Y el al. (2023) [59]	MIBC	236	Luminal, Basal	Luminal: GATA3, KRT20<br>Basal: KRT5/6, KRT14<br>Risk stratification in luminal tumours: YAP1, CCNB1	Basal tumours were associated with poorer prognosis. Two subgroups in luminal MIBC were identified based on YAP1 and CCNB1 expression, where low expression of these markers indicated poorer prognosis.	Molecular subtyping using IHC is a practical and cost-effective method to predict prognosis in MIBC. The use of YAP1 and CCNB1 allows further risk stratification within the luminal MIBC cohort
Koll FJ et al. (2022) [38]	MIBC	181	Basal, Luminal, DN	KRT5/6, GATA3	KRT5/6 was associated with the basal subtype, GATA3 with the luminal subtype. Double-negative cases showed greater molecular heterogeneity and worse prognosis.	IHC-based classification is associated with histological subtypes of MIBC, but a more complex classification system may be needed to

						reflect the disease's heterogeneity and guide therapeutic decisions.
Olkhov-Mistel E et al. (2022) [50]	MIBC	243	Uro, GU, basal	KRT5/6, GATA3, p16	he three-antibody IHC panel (KRT5/6, GATA3, p16) effectively classified MIBCs into luminal and basal subtypes. Luminal cases were predominant (78.8%), with 21.2% basal. Basal subtypes were associated with divergent differentiation and worse disease-specific survival. Within luminal subtypes, GU tumours had higher abnormal p53 staining (80.2%) compared to URO (38.1%). The panel demonstrates clinical potential for distinguishing MIBC subtypes with prognostic implications.	This retrospective study demonstrates the potential utility of a three-antibody IHC panel to differentiate luminal and basal MIBC.
Wang T et al. (2022) [90]	NMIBC	160	Luminal (Class 2), Basal (Class 3), Mixed (Class 1)	Uroplakin2, KRT20, KRT5/6, CD44	Molecular classification based on IHC predicts the efficacy of intravesical chemotherapy. Gemcitabine reduces recurrence more effectively in basal-like subtypes compared to anthracyclines.	The study demonstrates that molecular subtypes identified through IHC are independent prognostic factors for NMIBC, and the efficacy of intravesical chemotherapy varies by subtype.
Queipo FJ et al. (2022) [51]	MIBC	113	URO, GU, Ba/Sq, Mes-like, NE-like	KRT5/6, KRT14, GATA3, p16	IHC-based subtyping classified 72.57% as luminal and 27.43% as non-luminal. Specific IHC markers effectively distinguished between subtypes.	IHC subtyping of MIBC using common markers is feasible and can guide treatment decisions in clinical practice. Validation in external cohorts is required.
Hardy CSC et al. (2022) [44]	MIBC	193 (Lund 2017 cohort), 76 (Lund 2012 cohort)	Basal, GU, URO	GATA3, KRT5/6, KRT14 p16, RB1	Simple IHC classifiers (GATA3, KRT5, p16) can accurately classify basal, Uro, and GU subtypes with 78%-86% accuracy. Models using KRT14 and RB1 showed similar accuracies.	Simple IHC classifiers validated by mRNA analysis can be implemented in clinical practice for accurate subtyping of MIBC.
Pryma C et al. (2022) [63]	MIBC	80	Basal and Luminal	KRT5/6, GATA3, Uroplakin2	Uroplakin2 was found to be a highly accurate single marker for distinguishing luminal from basal subtypes.	Uroplakin 2 with a staining cutoff of 50%, can reliably classify MIBC into luminal and basal subtypes.
Jackson CL et al. (2022) [80]	NMBC	481	Basal, GU, URO, URO-KRT5+	GATA3, KRT5/6, p16	The three-antibody algorithm effectively classifies NMIBC into four subtypes, with significant prognostic implications, particularly in identifying low-risk URO-KRT5+ and high-risk basal subtypes.	The algorithm can assist in patient stratification for NMIBC, identifying those at low or high risk of recurrence and progression, and informing treatment strategies.
Bai Y et al. (2022) [58]	MIBC	119	Luminal, Basal, DN	CD44, KRT5/6, KR20, PDL1, p53	Computer-aided diagnosis system based on pathological images demonstrated high accuracy in predicting molecular subtypes (94% accuracy), PD-L1 status (89.7% accuracy), and p53 status (84.6% accuracy).	The system can reduce the workload of pathologists and expedite the identification of molecular subtypes and the status of PD-L1 and p53, potentially improving personalised treatment in bladder cancer.
Bernardo C et al. (2022) [46]	MIBC	347	Ba/Sq, Sc/NE, Uro A, Uro B, GU	KRT5, KRT14, PPARG, GATA3, CCND1, CDKN2A, RB1	Ba/Sq tumours show diffuse KRT5 expression and lack of differentiation markers. Sc/NE tumours show high proliferation and lack of differentiation similar to GU.	IHC patterns clearly distinguish Ba/Sq from UroB and Sc/NE from GU, confirming that these are distinct molecular subtypes with different phenotypic features.
Muilwijk T et al. (2021) [81]	NMIBC	109	Not explicitly classified as molecular subtypes but focused on basal and luminal markers	KRT5/6, GATA3, p40, p63	Identifies a correlation between KRT5 and KRT20 expression with tumour grade in NMIBC, suggesting their potential role in differentiating high-grade from low-grade disease.	The study found an inverse correlation between KRT5 expression and tumour grade, and a positive correlation between KRT20 expression and high-grade disease, indicating distinct biological behaviours in NMIBC compared to MIBC.

Haghayeghi K et al. (2021) [61]	MIBC	43	Basal, Luminal and Indeterminate	KRT5/6, CD44, KRT14, GATA3, KRT20, HER-2, Ki67	The study categorises MIBC into luminal and basal subtypes based on IHC markers and correlates these subtypes with distinct patterns of muscularis propria invasion. Pattern 1 (encasing muscle bundles) is associated with luminal phenotype, while Pattern 2 (dissecting muscle) is linked with basal phenotype and higher aggression. p53-Wild-Type phenotype is more common in the basal subtype.	A panel of KRT20 and GATA3 for luminal subtypes and KRT5/6 and KRT14 for basal subtypes can effectively classify molecular subtypes in MIBC. The morphology of muscularis propria invasion in MIBC can predict molecular subtype and tumour behaviour, with basal subtypes linked to more aggressive features and poorer outc
Lu J et al. (2021) [89]	NMIBC	176	Luminal A, Luminal B, BasalQ	Uroplakin2, GATA3, KRT14, KRT 5/6, KRT20, CD44	Luminal A: High expression of KRT20 and Uroplakin II, low expression of KRT5/6, KRT14, CD44, and GATA3. Luminal B: High expression of GATA3 and Uroplakin II, low expression of KRT5/6, KRT14, CD44, and CK20. Luminal A showed better RFS and PFS. BASQ tumours showed better PFS with GC intra-arterial chemotherapy than with intavesical chemotherapy.	IHC subtypes predict chemotherapy response and survival in T1 NMIBC. IHC subtyping may guide personalised therapy.
Bontoux C et al. (2021) [74]	MIBC	187	Luminal, Ba/sq	GATA3, FOXA1, KRT5/6, KRT14	IHC panel distinguished between Luminal and Basal/Squamous subtypes. Luminal tumours were (GATA3 /FOXA1+) and Basal/Squamous were (KRT5/6 /KRT14+). High concordance between primary tumours and Lymph Node Metastasis	A four-antibody IHC panel is effective for UC subtyping and assessing metastatic potential. Further validation needed.
Ikeda J et al. (2021) [53]	MIBC	106	Luminal, Basal	KRT5/6, KRT14, KRT20, GATA3, Uroplakin II	High correlation between basal subtypes with elevated TAICs. TAICs significantly discriminate cancer-specific survival	Combined evaluation of histological, molecular subtypes, and TAICs may guide prognosis and treatment. Comprehensive pathological evaluation is feasible and impacts oncological outcomes.
Garcyk S et al. (2021) [87]	NMIBC	128	Luminal, Null, Mixed	KRT20, KRT5/6, KRT14, GATA3, p53, ERBB2	IHC analysis of surrogate molecular subtypes in high-risk NMIBC with CIS; evaluation of inter-lesional heterogeneity. Significant intratumoural heterogeneity of CIS lesions; patient age and smoking status identified as prognostic factors.	Further study on the heterogeneity of surrogate molecular subtypes is needed for better prognostic stratification.
Bejrananda T et al. (2021) [56]	MIBC	132	Luminal-like, Basal-like, Mixed, DN	GATA3, KRT5/6, KRT14, KRT20	DN subtype (GATA3-/KRT5/6-) had the worst prognosis with a 5-year OS of 7.14%. GATA3 and KRT5/6 expression were significantly associated with survival outcomes.	HC subtyping using GATA3 and KRT5/6 can help identify patients with poor prognosis who may benefit from more intensive therapy.
Mandelli GE et al. (2020) [62]	MIBC	84	Basal and Luminal	KRT5/6, KRT14, KRT20, Uroplakina 2	Higher density of CD66b+ TANs in basal-type MIBC; significant correlation with STAT3 activation and FOSL1 expression	Basal-type UBCs are associated with dense infiltration of TANs, linked to a pro-inflammatory environment; potential relevance for patient stratification in immunotherapy
Font A et al. (2020) [70]	MIBC	126	Basal, Luminal, Mixed	GATA3, KRT5/6, KRT14, FOXA1	BASQ-like tumours (FOXA1/GATA3 low, KRT5/6/14 high) are more likely to achieve pCR to NAC (OR = 4.06), with no significant survival difference compared to luminal tumours.	IHC-based classification can be used to identify MIBC patients who are more likely to benefit from NAC, particularly those with BASQ-like tumours.
Guo CC et al. (2020) [37]	MIBC	TCGA cohort (n=408), MDAnders on cohort (n=221)	Luminal, Basal, Double-negative	Uroplakin2, GATA3, KRT14, KRT 5/6, KRT20	Developed BLT score to quantify and identify molecular subtypes; analysis of EMT status and immune profile	Immunohistochemical classifier (GATA3, KRT5/6) can reliably identify molecular subtypes in clinical practice

Ottley EC et al (2020) [91]	NMIBC	26	Luminal, basal, EMT	KRT5/6, KRT14, CD44, GATA3, FOXA1, KRT20, Vimentin, E-cad, , N-cad, Slug/snail, Axl	Luminal Markers FOXA1 and SCUBE2 were significantly associated with better disease-specific survival. The markers related to the EMT process were not significant predictors of disease-specific survival.	The molecular subtypes analysed through immunohistochemistry are better predictors of prognosis than the markers of the EMT process in high grade T1 NMIBC.
Rebola J et al. (2019) [77]	NMBIC	147	Luminal, Basal, Mixed, Null	KRT20, KRT5/6	Luminal subtype associated with poorer survival outcomes. Basal subtype shows intermediate prognosis, while null and mixed subtypes are less aggressive.	IHC-based molecular classification (luminal/basal) is a significant predictor of prognosis in NMIBC, with the luminal subtype linked to more aggressive disease. Further validation is needed for routine clinical use.
Shelekova et al. (2019) [52]	NMIBC	49	UroA, UroD, GU, Ba/Sq, Sc/NE	KRT5/6, KRT14, KRT20, p16, E2F3, FOXA1, RB1, Ki67	Urothelial carcinoma in young patients predominantly classified as UroA subtype with favourable prognosis. Uro D subtype exhibited higher p16, E2F3, Ki67, and KRT14 expression. Aggressive subtypes such as GU, Ba/Sq-like, and Mes-like were rare and seen in older patients.	The majority of young patients exhibit the UroA molecular subtype, associated with good prognosis. More aggressive subtypes become more frequent with age.
Jangir H et al. (2019) [57]	MIBC	40	Basal , Luminal, Dual, Null	GATA3, KRT5/6, KRT14, KRT20	IHC-basal MIBCs showed poorer survival outcomes compared to luminal and dual subtypes. GATA3 loss and KRT14 positivity were associated with the worst prognosis.	A simple IHC panel including GATA3 and KRT14 can effectively stratify MIBC patients into prognostic groups, providing a basis for targeted patient management.
Bernardo C et al. (2019) [45]	MIBC/NMIBC	344	UroA, UroAp, UroB, UroC, GU	KRT5, EGFR, CDH3, FGFR3, ERBB2, CCND1, RB1, p16, p63, KRT20, Uroplakina3	Uro tumours were characterised by high FGFR3, CCND1, and RB1 expression, low or absent p16 and ERBB2 expression. UroC resembles GU in ERBB2 expression but is closer to UroA in FGFR3 expression. GU tumours are distinct with loss of basal markers, high ERBB2, and low FGFR3 expression.	Uro tumours were characterised by high FGFR3, CCND1, and RB1 expression, low or absent p16 and ERBB2 expression. UroC resembles GU in ERBB2 expression but is closer to UroA in FGFR3 expression. GU tumours are distinct with loss of basal markers, high ERBB2, and low FGFR3 expression.
Rodriguez Pena et al. (2019) [79]	NMIBC	60	Basal and Luminal	KRT5/6, KRT20, Uroplakin 2, CD44, GATA3, HER2/neu, ER	CD44 associated with recurrence; HER2/neu associated with less grade progression.	Individual IHC markers may be of prognostic value in NMIBC. Further studies needed to validate findings.
Barth I et al. (2018) [88]	NMIBC/MIBC	156	Basal and Luminal	KRT20, GATA3, HER2/neu, ERβ, KRT5/6, KRT14, p53	Majority of CIS cases express luminal markers and show a switch to basal markers during progression to MIBC.	IHC used to track the transition from luminal to basal phenotypes during progression from CIS to invasive cancer. ERβ and Her2 may be potential targets for new therapies in CIS.
Sjödahl et al. (2018) [42]	MIBC	67	UroA, UroB, UroC, GU, Ba/Sq, Sc/NE	29 markers (KRT5, GATA3, ERBB3, TP63, CDH1, etc.)	Cohort of MIBC metastatic in lymph node with high concordance in luminal subtypes between primary tumours and metastases. Significant discordance in the Basal/Squamous subtype. Intra-tumour heterogeneity was common.	IHC-based molecular classification shows high concordance, but heterogeneity and discordance in Basal/Squamous subtypes suggest caution when using IHC for therapeutic planning in MIBC metastatic in lymph node.
Sjödahl et al. (2017) [28]	MIBC	307	URO, GU, Ba/Sq-like, Mes-like, Sc/NE	CCNB1, CCND1, CDH1, CDH3, p16, CHGA, E2F3, EPCAM, FGFR3, FOXA1, GATA3, KRT5, KRT14, KRT20, NCAM1, PPARG, RB1, RXRA, SYP, TP63, TUBB2B, UPK3, VIM, ZEB2	Identified five tumour-cell phenotypes in advanced urothelial carcinoma: Uro, Ba/Sq-like, Mes-like, Sc/NE	Systematic disagreement between global mRNA profiling and tumour-cell phenotype by IHC. A combined classification is suggested
Xiao GQ et al. (2017) [60]	MIBC	22	Luminal, Basal, Dual, Null	KRT5, KRT14, KRT20, Her2/Neu, p53	Basal subtype was more common in females; high Her2/Neu overexpression in luminal and dual subtypes; p53 expression was consistent across Urothelial Carcinoma Small Cell Carcinoma	Identification of molecular subtypes can guide targeted therapies, such as Herceptin for Her2/Neu-positive urothelial carcinoma

Dadhania V et al. (2016) [20]	NMIBC/MIBC	MDAnderson (n=132), Lund (n=308), TCGA (n=408), MD Anderson FFPE (n=89)	Luminal, Basal	GATA3, KRT20, KRT18, Uroplakin 2, Cyclin D1, ERBB2/HER2, KRT5/6, KRT14, p63, BCL2, Smooth muscle actin, Myosin, Calponin, Desmin	Bladder cancers can be consistently classified into luminal and basal subtypes with distinct clinical behaviours. The use of GATA3 and KRT5/6 markers alone could classify tumours with over 90% accuracy.	The study confirmed that bladder cancer subtypes have distinct clinical behaviours, with basal tumours being more aggressive. A two-marker IHC classifier using GATA3 and KRT5/6 was effective for prognostic and therapeutic stratification

Ba/Sq: Basal/Squamous; BLT: basal to luminal transition; DFS: disease-free survival; DN: double-negative; DP: double positive; EMT: epithelial-mesenchymal transition; GU: genomically unstable; LumP: luminal papillary; Mes-like: mesenchymal-like; NE-like: neuroendocrine-like; OS: overall survival; PFS: progression-free survival; RFS: recurrence-free survival; Sc/NE: Small Cell/Neuroendocrine-like; TAICs: tumour-associated immune cells; TANs: Tumour-Associated Neutrophils; URO: urothelial-like
